# Study of Histomorphological Patterns of Uterine Leiomyomas: An Observational Study

**DOI:** 10.31729/jnma.8883

**Published:** 2025-02-28

**Authors:** Archana Tiwari, Pratima Sapkota

**Affiliations:** 1 Department of Pathology, Lumbini Medical College, Tansen, Palpa, Nepal

**Keywords:** *degeneration*, *fibroids*, *histopathology*, *hysterectomy*, *leiomyoma*

## Abstract

**Introduction:**

Uterine leiomyoma, also known as a fibroid, is a benign mesenchymal tumor derived from the smooth muscle of the uterus. It is the most common tumor in women with an estimated incidence of 20%-40% in women during their reproductive years. Leiomyoma can occur in any organ, but the most common forms appear in the uterus. This study is conducted to analyze histomorphological patterns of uterine leiomyomas.

**Methods:**

An observational cross-section was conducted between 1st June 2021 and 31st May 2023 in the Department of Pathology of a Medical College and Teaching Hospital. Patients with leiomyoma were included in the study. Clinical, sonographic, gross, and histopathology findings were analyzed. Ethical approval for the study was obtained from Institutional Review Committee (Reference Number: LMC10/B-021).

**Results:**

There were 100 patients with leiomyoma during the study period, among whom 55 (55%) were 40-49 years old. There were 65 (65%) patients in the group with a parity of two to three. Abnormal uterine bleeding was observed in 60 (60%) of the cases. Histologically 84 (84%) of the cases were simple conventional leiomyoma. The co-existing conditions found were cystic ovarian disease 37 (41.11%), adenomyosis 19 (21.11%), cervical intra epithelial neoplasia 12(13.33%).

**Conclusions:**

Conventional leiomyoma is the commonest histological subtype and and the most common clinical presentation is abnormal uterine bleeding.

## INTRODUCTION

Leiomyoma is the most common benign uterine tumor in women of reproductive age group caused by hyperestrogenism.^1,2^ The histopathological and clinical presentation of fibroids is still obscure, requiring further research by pathologists.^3^ Advanced technologies have confirmed genetic alterations in conversion of Myometrial Stem Cells.^4^ Wide morphologic spectrum of leiomyomas poses diagnostic problems. ^5^

Microscopically, leiomyomas show fascicles of smooth muscles with frequent degenerative changes.^6,7^ Fibroid have variable sizes and numbers however they are not directly related to presenting symptoms.^8^

Menorrhagia is the most common presenting symptom.^9^ Abnormal Uterine Bleeding (AUB) is the leading cause of elective hysterectomy.^10^

To guide focused therapeutic strategies, a thorough understanding of the pathology and histology connected to Leiomyoma is crucial.^11^ This study aims to find the clinical presentation and histopathological subtypes of leiomyoma.

## METHODS

This is an observational cross-section study conducted at the Department of Pathology, Lumbini Medical College and Teaching Hospital (LMCTH), a tertiary care centre at located at Palpa, Nepal. All patient visiting the hospital from 1^st^ June 2021 to 31^st^ May 2023 undergoing surgical management for leiomyoma were considered as the population for the study. Ethical approval was taken from the Institutional Review Committee (Reference number: LMC 10/B-021). Written informed consent was taken from all participants at Department of Pathology, LMCTH. Patients above 18 years of age were included and the patients not agreeing to give consent were excluded from the study.

Patient's age, parity, clinical presentation were recorded from the requistion form , gross (number, and locations), and histopathology findings were recorded at histopathology lab. The hysterectomy and myomectomy specimens were formalin fixed and examined for the location, number, and gross morphology. Representative sections were submitted for evaluation. Paraffin embedded sections were cut at 3-5-micron on microtome and stained with Hematoxylin & Eosin stain. Microscopic examination and reporting were done. Data were entered in Microsoft Excel and descriptive analysis was done.

## RESULTS

**Figure 1:**
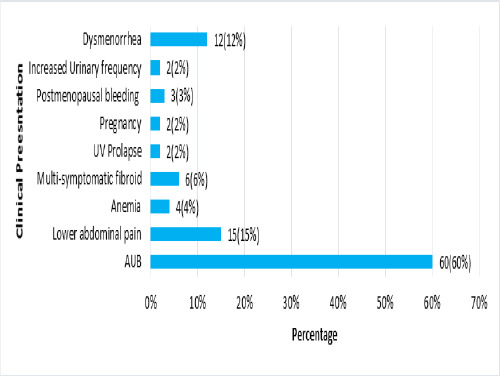
Clinical presentation of a patient with uterine leiomyoma (n=100).

There were 100 participants undergoing surgical managment for leiomyoma. The mean age was 44.95 years (±7.36). There were no patients of age below 20 years, 6 (6%) patients between the age of 20-30 years and 94 (94%) patients between the age of 30-60 years. Among the total patients, 18 (18%) were at first parity, 35 (35%) were at second parity, 30 (30%) were at third parity and 17 (17%) were grand multiparous. Uterine Bleeding was observed in 60 (60%) patients (Figure 1).

Uterine corpus was involved in 96 (96%) cases, cervical fibroid in 2 (2%) and ligament fibroid in 2 (2%) cases. Multiple leiomyomas were noted in 54 (54 %) cases and 46 (46 %) had solitary leiomyoma. Hysterectomy (Total Abdominal Hysterectomy with Bilateral Salphingo-oophorectomy was done in 90 (90%) cases and 10 (10 %) cases had myomectomy. Intramural, sub-serosal, submucosal and cervical leiomyoma were 63 (63%), 34 (34%), 17 (17 %), 2 (2%) respectively. Broad and round ligament leiomyoma were total of 2 (2%); 1(1%) each.

The median size of fibroid was calculated as 4.75cm (IQR: 0.125-9.375cm) ranging from 0.3 cm to 22cm in size. The size of fibroid in 44 (44%) cases was between 1-5cm, and 6-10 cm in 37 (37%) cases.

**Figure 2:**
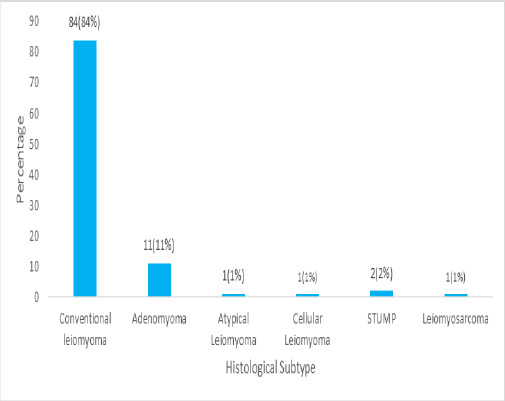
Histological subtypes of uterine leiomyoma (n=100).

Histopathological findings revealed that 84 (84%) cases were simple conventional leiomyoma (Figure 2). Among the cases of Adenomyomas, only 3 (75%) had associated adenomyosis and 1 (25%) had associated ovarian endometriosis.

Among the uterus with degenerative changes, 26 (26%) had hyaline degeneration (Table 1). Hyaline degeneration and cystic degeneration alone made 31 (86.11%) of degenerative changes in leiomyoma. Of the endometrium of hysterectomized patients, disordered proliferative endometrium, proliferative phase of endometrium, and hyperplasia without atypia in aggregate made 69 (66.67%) of the endometrial pattern.

**Table 1:** Degenerative changes in uterine leiomyoma (n=36).

Type of degeneration	n (%)
Hyaline degeneration	26(72.22)
Cystic degeneration	5(13.89)
Calcification	4(11.11)
Hydropic degeneration	1(2.28)

**Table 2:** Endometrial pattern in hysterectomized uterus (n=90).

Endometrial pattern	n (%)
Proliferative phase	26(28.89)
Disordered Proliferative endometrium	28(31.11)
Secretory phase	15(16.67)
Hyperplasia without atypia	6(6.67)
Gestational endometrium	1(1.11)
Atrophic endometrium	4(4.44)
Chronic endometritis	8(8.89)
Hormone induced change	2(2.22)

**Table 3:** Co-existing clinical pathology in female who underwent hysterectomy (n=90).

Uterine pathology	n (%)
Chronic cervicitis	90(100)
Cystic ovaries	37(41.11)
Adenomyosis	19(21.11)
Cervical intraepithelial neoplasia (CIN)	12(13.33)
Endometriosis/endometriotic cyst	10(11.11)
Benign serous cystadenoma	3(3.33)
Cervical polyp	3(3.33)
Endometrial polyp	2(2.22)

Disordered proliferative endometrium (DPE) was found in 28 (31.11%) cases and proliferative phase of endometrium in 26 (28.89%) cases (Table 2).

Chronic cervicitis was seen in 90 (100%) cases (Table 3).

## DISCUSSION

Uterine leiomyomas are the most common gynecologic neoplasm and hysterectomy is the frequent surgery performed for Leiomyoma and related pathologies. ^1,2^ Uterine leiomyoma shows a wide spectrum of morphologies and clinicopathological features.

The present study included 100 cases of uterine fibroid in which the incidence of fibroids was highest for two decades of reproductive years in the age range of 40 to 59 years, comprising of 75 (75%) of the cases, among which 55 (55%) were in 40-49 years of age group. This was in accordance with various studies across the globe. Kaushal A et al. ^9^ revealed age group of 5060 years being most common 49 (45%), however, 2nd most common age group was 41-50 years comprising of 46 (42.2%). Yamuna et al. ^1^ shows highest incidence at age of 36-45years with 64 (64%) within this age group. Ghazaly et al. ^3^ showed 61 (50.8%) of cases in < 35 years of age group and 59 (49.2%). cases in ≥ 35 years age group which is discordant with our study. Mean age of 39 years was seen Kulkarni et al. ^2^ which is different from our study with mean age of 44.95 years. Those aged 41-50 or 51-60 years were 10 times more likely to have leiomyoms than those aged 21-30 years according to Steward et al.^12^

Majority of the cases in this study presented with leiomyomas had parity between 2-3 in our study 65 (65%). Parity of 3-5 was seen in 24 (48%) cases in Ara et al. ^10^

Abnormal uterine bleeding was the most common presentation in our study 60 (60%). Dysmenorrhea in 12(12%). This is similar to Kaushal et al. ^9^ with AUB seen in 78(71.6%) cases and dysmenorrhea in 20 (18.3%) and Yang et al. ^4^ in which AUB is seen in 76% and dysmenorrhea in 20%. However, AUB was seen in only 36(30%) cases in Khan et al ^13^. AUB remained the most common clinical presentation throughout various studies.

Size of leiomyoma of > 5cm is seen in 50 (50%) of the cases in our study. 54 (54%) of patients have multiple fibroids. Ghazaly et al.^3^ had a slightly lower percentage 46 (38.3%) of patients with size larger than 5cm. However, this study varies from our current study as solitary fibroid were the commonest 88 (73.3%).

Intramural fibroid is seen in 63 (63%) of the cases in our study and submucosal in 17 (17%) of cases. Khan et al. ^13^ had similar findings with intramural fibroid being in 81 (67.50%) of cases and 5 (4.16%) had submucosal fibroid. Similar presentation was in Kulkarni et al. ^2^ with intramural fibroid in 60 (60.6%) cases and 9 (9.1%) with submucosal fibroid. One of the study done by Ara et al^10^ had only 16 (32.7%) intramural and subserosal fibroid.

Disordered proliferative endometrium 28 (31.11%), and proliferative phase of endometrium 26 (28.29%) was the commonest presentation. This is different from most of the study as 77 (70.6%) of cases had proliferative phase of endometrium in Kaushal et al.^9^ 74 (61.66)% of proliferative phase of endometrium is seen in Khan et al^13^ with only 5 (4.16%) having DPE. 68 (68%) had proliferative phase of endometrium is in Yamuna et al^1^. DPE and other proliferative pattern are indicative of an anovulatory cycle which could be possible cause of hyperestrogenism.

Degenerative changes was seen in 36 (36%) of cases with hyaline degeneration 26 (26%) and cystic degeneration 5 (5%) being the commonest and 4 (4%) had calcareous degeneration. This is in contrast with Ara et al.^10^ where only 2 (4%) had degenerative changes. But our study was concordant with Kaushal A et al.^9^ with 22 (20.2%) hyaline degeneration and 5 (4.6%) cystic degeneration, and Khan et al.^13^ had only 22 (18.33%) degenerative change with 15 (12.5%) being hyaline degeneration. Another study with even higher degenerative changes was seen in Naaz et al.^14^ which showed 56 (58.61%) hyaline degeneration and 47 (4.87%) who had cystic degeneration. Hyaline degeneration was the most common type of degeneration, accounting for 60% of cases as per Raubenheimer et al^15^ showing concordance with our study. High percentage of degenerative changes in uterine fibroid in our study could be because of prolonged duration of untreated fibroid or undiagnosed fibroid with late presentation.

Molecular parameters, immunohistochemical profile and hormonal status of the patient couldn't be included in the study and so was not compared with other literatures.

This study has several limitations that need to be considered. The cross-sectional design limits the ability to establish causal relationships or assess changes over time in the pathology of leiomyomas. The study was conducted in a single tertiary care centre, which may restrict the applicability of findings to other populations with different demographic and clinical characteristics. Molecular and immunohistochemical analyses, as well as hormonal profiling, were not included, which could have provided more detailed insights into the causes and progression of leiomyomas. The sample size of 100 cases, while sufficient for descriptive analysis, may not adequately represent rare subtypes or variations in leiomyoma pathology. Additionally, the absence of a control group limits comparative analysis with normal uterine tissue, thereby narrowing the scope of the findings to fibroid pathology alone.

## CONCLUSIONS

The conventional type was the most common uterine leiomyoma observed in this study. Abnormal uterine bleeding was the most common clinical presentation and hyaline degeneration was the most common degenerative change. The disordered proliferative endometrium was observed the most, and all 90 hysterectomized uterus showed evidence of chronic cervicitis.
